# Promiscuous Defluorinating Enoyl-CoA Hydratases/Hydrolases Allow for Complete Anaerobic Degradation of 2-Fluorobenzoate

**DOI:** 10.3389/fmicb.2017.02579

**Published:** 2017-12-21

**Authors:** Oliver Tiedt, Mario Mergelsberg, Wolfgang Eisenreich, Matthias Boll

**Affiliations:** ^1^Faculty of Biology – Microbiology, Institute of Biology II, University of Freiburg, Freiburg, Germany; ^2^Lehrstuhl für Biochemie, Technische Universität München, Munich, Germany

**Keywords:** enzymatic defluorination, fluoroaromatics, enoyl-CoA hydratase, benzoyl-CoA reductase, anaerobic aromatic catabolism

## Abstract

Biodegradation of the environmentally hazardous fluoroaromatics has mainly been associated with oxygenase-dependent defluorination reactions. Only very recently a novel mode of oxygen-independent defluorination was identified for the complete degradation of *para*-substituted fluoroaromatics in the denitrifying *Thauera aromatica*: a promiscuous class I benzoyl-coenzyme A (BzCoA) reductase (BCR) catalyzed the ATP-dependent defluorination of 4-F-BzCoA to BzCoA. Here, we studied the unknown enzymatic defluorination during the complete degradation of 2-F-benzoate to CO_2_ and HF. We demonstrate that after activation of 2-F-benzoate by a promiscuous AMP-forming benzoate-CoA ligase, the 2-F-BzCoA formed is subsequently dearomatized by BCR to a mixture of 2-F- and 6-F-cyclohexa-1,5-diene-1-carboxyl-CoA (2-F-/6-F-1,5-dienoyl-CoA). This finding indicates that BCR is not involved in C–F-bond cleavage during growth with 2-F-benzoate. Instead, we identified defluorination of the two isomers by enoyl-CoA hydratases/hydrolases involved in down-stream reactions of the BzCoA degradation pathway. (i) The 1,5-dienoyl-CoA hydratase hydrated the F-1,5-dienoyl-CoA isomers to a mixture of the stable 2-F-6-OH-1-enoyl-CoA and the unstable α-fluorohydrin 6-F-6-OH-1-enoyl-CoA; the latter spontaneously decomposed to HF and 6-oxo-cyclohex-1-enoyl-CoA (6-oxo-1-enoyl-CoA), a common intermediate of the BzCoA degradation pathway. (ii) 6-Oxo-1-enoyl-CoA hydrolase/hydratase catalyzed the defluorination of 2-F-6-OH-1-enoyl-CoA to 2-oxo-6-OH-1-enoyl-CoA and HF again via water addition to an F-enoyl-CoA functionality. Based on these *in vitro* results, we demonstrate a previously overseen capability of 2-F-benzoate degradation for many but not all tested facultatively and obligately anaerobic bacteria that degrade aromatic compounds via the BzCoA degradation pathway. In conclusion, the newly identified enzymatic defluorination by enoyl-CoA hydratases via α-fluorohydrin formation represents an abundant, physiologically relevant principle of enzymatic defluorination.

## Introduction

In the last decades fluorinated organic compounds have become relevant environmental contaminants formed by industrial, agricultural and pharmaceutical processes. Among them, stable and recalcitrant fluoroaromatics are of concern (Key et al., 1997; Park et al., 2001; Smart, 2001; Weinstein and Davison, 2005; Murphy et al., 2008, 2009; Purser et al., 2008; Zhang et al., 2012). Despite originally perceived as biologically inert, fluoroaromatics are meanwhile known to serve as growth substrate for aerobic and anaerobic bacteria that play an important role in the elimination of fluoroaromatics from the environment. Fluorobenzoates (F-benzoates) are the best studied fluoroaromatic model compounds for microbial degradation (Murphy et al., 2009; Murphy, 2010; Kiel and Engesser, 2015; Tiedt et al., 2016).

Some aerobically growing microorganisms of the genera *Agrobacterium, Pseudomonas, Alcaligenes, Aureobacterium, Thauera* and others use the 2-, 3- and 4-F-benzoate isomers as growth substrates (Kiel and Engesser, 2015). In the case of 2-F-benzoate degradation, promiscuous ring-hydroxylating dioxygenases form fluorinated dihydrodiol intermediates, which, depending on the isomer formed, are prone to spontaneous elimination of the fluorine substituent. In contrast, defluorination of 3-/4-F-benzoate may be accomplished on the level of 4-fluoromuconolactone. Mechanistic considerations for spontaneous or enzyme catalyzed fluorine release at this stage are on debate (Natarajan et al., 2005; Murphy, 2010; Kiel and Engesser, 2015).

Though there are several reports on the complete degradation of 2-F-, 4-F- and 3-Cl benzoate by denitrifying bacteria, much less is known about the enzymology of the dehalogenation reactions involved in the complete degradation of fluoroaromatics in the absence of oxygen. The typical process of oxygen-independent dehalogenation in bacteria is organohalide respiration, where organohalides generally serve as terminal electron acceptors in respiratory chains but usually not as source of carbon and electrons. Here, the carbon-halide bond is cleaved by corrinoid containing reductive dehalogenases (Holliger and Schumacher, 1994; Hug et al., 2013; Jugder et al., 2016). In spite of the general importance of organohalide respiration for the anaerobic degradation of organochlorides and -bromides, the reaction involved has never been observed with fluorinated compounds probably owing the strength of the C–F-bond (Cooper et al., 2015).

Only recently, first insights in the enzymatic processes involved in C–halide bond cleavage during complete degradation of haloaromatic compounds without oxygen have been obtained. The degradation of 3-Cl-benzoate is initiated by activation to its CoA thioester by a specific ligase. The 3-chlorobenzoyl-CoA (3-Cl-BzCoA) formed is then dearomatized by ATP-dependent BzCoA reductase (BCR) to 3-Cl-cyclohexa-1,5-diene-1-carboxyl-CoA (3-Cl-1,5-dienoyl-CoA), that spontaneously eliminates HCl driven by aromatization to BzCoA (Egland et al., 2001; Kuntze et al., 2011). In contrast, 3-F-BzCoA is reduced to a much more stable 3-F-1,5-dienoyl-CoA dead-end product, which is considered the reason why growth with 3-F-benzoate has never been observed with an anaerobic organism.

**FIGURE 1:**
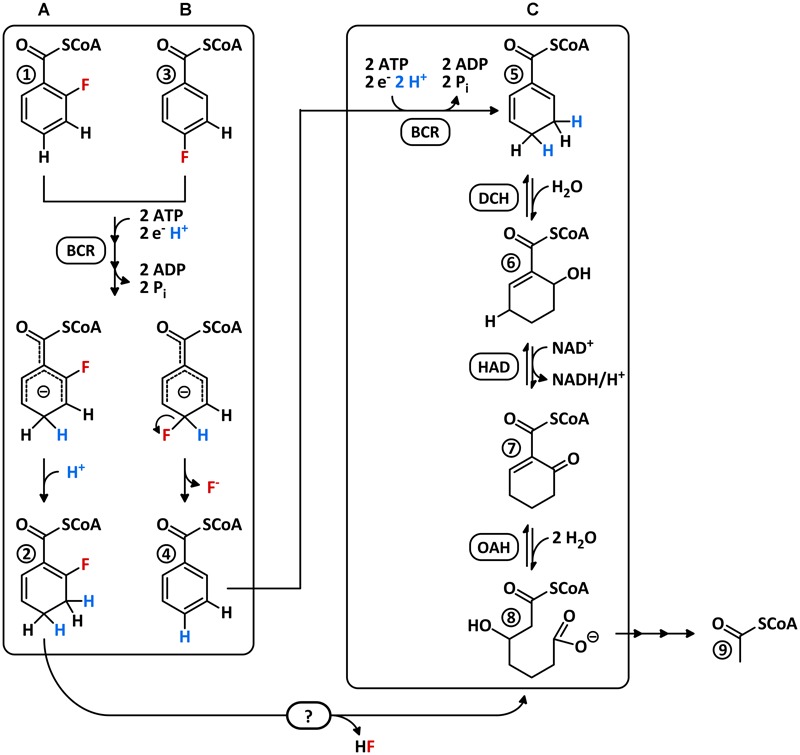
Scenarios for the catabolism of 2- and 4-F-BzCoA via the BzCoA degradation pathway. Putative conversion of **(A)** 2-F-BzCoA to F-1,5-dienoyl-CoA and **(B)** experimentally verified defluorination 4-F-BzCoA to BzCoA by class I BCR. Both compounds are suggested to be ATP-dependently reduced to a common anionic transition state, which is either protonated at C3/C5 to a 2- or 6-F-1,5-dienoyl-CoA **(A)** (only the 2-F-isomer is shown) or defluorinated to BzCoA **(B)** at the *para*-position. Direct defluorination of 2-F-BzCoA by BCR is unlikely as it would require protonation at C2 instead of C4. **(C)** BzCoA degradation pathway involving ATP-dependent BCR, DCH, HAD, and OAH. At the beginning of this study it was unknown how 2-F-BzCoA is channeled into the BzCoA degradation pathway.

Dehalogenation of a fluoroaromatic in the absence of oxygen was very recently reported for the denitrifying *Thauera aromatica* growing with 4-F-benzoate and nitrate (Tiedt et al., 2016). Similar to 3-Cl-benzoate, 4-F-benzoate is readily activated to the corresponding CoA thioester by promiscuous AMP-forming benzoate-CoA ligase (BCL). Then, promiscuous class I BCR catalyzes the electron donor- and ATP-dependent reduction of 4-F-BzCoA to BzCoA and HF. However, in contrast to 3-Cl-BzCoA dechlorination, a reduction/elimination mechanism is not feasible for 4-F-BzCoA defluorination. Instead, a reaction similar to nucleophilic aromatic substitution (S_N_Ar) at an anionic intermediary state was suggested, that is stable enough to permit C–F-bond cleavage (**Figure 1**).

The BzCoA formed after reductive 3-Cl- or 4-F-BzCoA dehalogenation is further metabolized by enzymes of the BzCoA degradation pathway. In denitrifying bacteria ATP-dependent class I BCRs dearomatize BzCoA to 1,5-dienoyl-CoA (**Figure 1**), followed by a 1,4-hydration to 6-hydroxycyclohex-1-ene-1-carboxyl-CoA (6-OH-1-enoyl-CoA) by 1,5-dienoyl-CoA hydratase (DCH) (Boll and Fuchs, 1995; Laempe et al., 1998; Thiele et al., 2008a). After oxidation of the latter to 6-oxo-1-enoyl-CoA by the NAD^+^-dependent alcohol dehydrogenase (HAD) (Laempe et al., 1999), the bifunctional 6-oxo-1-enoyl-CoA hydrolase (OAH) hydrates and hydrolyses the cyclic oxo-1-enoyl-CoA ring system to aliphatic 3-OH-pimeloyl-CoA (Laempe et al., 1999; Boll et al., 2000). Notably, both DCH and OAH represent unusual members of the highly divers crotonase-like superfamily and are only distantly related to other members of this family (Peters et al., 2007; Kuntze et al., 2008).

Growth with 2-F-benzoate has been described for some denitrifying *Thauera* and *Azoarcus* species (Schennen et al., 1985; Anders et al., 1995; Song et al., 2000; Mechichi et al., 2002). BCL readily activates 2-F-benzoate to 2-F-BzCoA at a rate similar to that of benzoate suggesting that 2-F-benzoate metabolism is initiated by CoA thioester formation (Schennen et al., 1985; Altenschmidt et al., 1991; Peters et al., 2004; Wischgoll et al., 2005). Moreover, BCR was reported to reduce 2-F-BzCoA even with a higher rate than BzCoA (Möbitz and Boll, 2002); however, the product was never identified, probably due to its instability. Notably, defluorination of 2-F-BzCoA by BCR via a S_N_Ar-like mechanism would require a sterically flexible proton donor at C2 (**Figure 1**).

In this work, we studied the unknown intermediates and enzymes involved in anaerobic 2-F-benzoate degradation using extracts of cells from *T. aromatica* grown with 2-F-benzoate and purified enzymes of the BzCoA degradation pathway. We identified a previously unknown mode of C–F-bond cleavage catalyzed by promiscuous enoyl-CoA hydratases/hydrolases of the common BzCoA degradation pathway that allows for growth with 2-F-benzoate.

## Materials and Methods

### Growth of Bacterial Cells and Preparation of Cell Extracts

Denitrifying strains were grown according to recent descriptions (Tiedt et al., 2016) with 2.5-fold molar excess of nitrate relative to substrate concentrations. Sulfate reducing strains were cultivated anaerobically at 30°C in a bicarbonate-buffered (30 mM) mineral salt medium containing the following components: 21.1 mM Na_2_SO_4_, 7.3 mM KH_2_PO_4_, 5.6 mM NH_4_Cl, 120 mM NaCl, 5.9 mM MgCl_2_, 6.7 mM KCl, 1 mM CaCl_2_, 2.25 mM Na_2_S, 23 μM Na_2_SeO_4_, 2 μM resazurin as well as each 1 mL L^-1^ of vitamin solution VL-7 (Pfennig, 1978) and of trace element solution SL9 (Tschech and Pfennig, 1984). Benzoate and 2-F-benzoate (1–2.7 mM) were used as the sole sources of carbon. Growth was monitored by measuring the optical density of cell suspensions in 1-cm cuvettes at 578 nm. Concentrations of growth substrates were determined as described recently (Tiedt et al., 2016).

### Cell Suspension Assays

Exponentially growing cells were harvested anaerobically by centrifugation (4,300 × *g*, 4°C, 15 min) and washed twice under equal conditions in mineral salt medium containing 12 mM NaNO_3_ but no carbon source. Cell suspensions were subsequently adjusted to an optical density of 9.0 (578 nm). Consumption reactions were started at 30°C by adding individual carbon substrates to a final concentration of 0.8 mM (final OD_578_ = 8.3) and stopped at individual time points by addition of equal volume amounts of 20% (vol/vol) formic acid. Samples were centrifuged at 18,000 × *g* (4°C, 10 min) prior to HPLC analysis of the supernatants by reversed-phase high-pressure liquid chromatography (HPLC) using a Waters 2690 separation module. A Eurospher 100-5 C18 column (250 by 4 mm) (Knauer) serving as solid phase was equilibrated with 40 mM formic acid containing 9% methanol at a flow rate of 1 mL min^-1^. After sample load, separation was achieved by a rising gradient of methanol to 45% within 2 min and further to 81% within 6 min. Individual substrate concentrations were determined by referencing to calibration standards.

### Synthesis and Analysis of CoA Thioesters

BzCoA was synthesized from benzoic acid anhydride and CoA (Schachter and Taggart, 1953) and halogenated analogs from the corresponding acids via their succinimide esters as described (Thiele et al., 2008b). The CoA ester metabolites 1,5-dienoyl-CoA and 6-oxo-1-enoyl-CoA were enzymatically produced from BzCoA using enriched enzymes of the anaerobic BzCoA degradation pathway as described earlier (Thiele et al., 2008b).

Reaction mixtures for enzymatic CoA thioester synthesis were treated with 2/3 of methanol (v/v, final content) and subsequent centrifugation for protein precipitation. Methanol was removed by flash evaporation at 40°C and the residual solution was freeze-dried. CoA thioesters were purified by preparative reversed-phase HPLC following previously described procedures (Thiele et al., 2008b). Desalting of purified compounds was achieved by solid phase extraction as described earlier (Erb et al., 2007).

Analysis of CoA thioesters was performed by reversed-phase ultraperformance liquid chromatography (UPLC^®^) on a Waters Acquity H-class system combined with a Eurospher II 100-2 C18 column (2.0 mm × 100 mm) (Knauer) at 30°C. For separation, acetonitrile content in a 10 mM potassium phosphate buffer (pH 6.8) was gradually increased at a flow rate of 0.23 mL min^-1^ from 2 to 12% within 2.4 min followed by 1.7 min up to 30%. This final concentration was kept constant for 1.1 min. Products were identified by comparing retention times and ultraviolet/visible (UV/vis) absorption spectra with standards and/or by subsequent mass spectrometric (MS) analysis. MS analysis was carried out by using a Waters Acquity I-class UPLC with a Waters C18 HSS T3 column (2.1 mm × 100 mm, 1.8 μm particle size) coupled to a Waters Synapt G2-Si HDMS ESI/Q-TOF system. For separation, a 7 min linear gradient from 2 to 30% acetonitrile in 10 mM ammonium acetate (pH 6.8) at a flow rate of 0.3 mL min^-1^ was applied. The mass spectrometer was operated in MS positive mode with a capillary voltage of 3 kV, 150°C source temperature, 450°C desolvation temperature, 1000 L h^-1^ N_2_ desolvation gas flow and 100 L h^-1^ N_2_ cone gas flow.

### Determination of Enzyme Activities

For determination of enzyme activities, a discontinuous HPLC-/UPLC-based assay was applied as described earlier (Boll et al., 2000). Identification and analysis of CoA thioester intermediates was routinely performed by C18 reversed phase UPLC and LC/MS as described above.

### Analysis of Structural Isomers by NMR Spectroscopy

Reaction products of 2- and 3-F-BzCoA with BCR were prepared for NMR spectroscopy as described above. The compounds were dissolved in 0.5 mL deuterated water. ^1^H NMR and ^13^C NMR spectra were recorded at 500 and 126 MHz, respectively, with Avance-HD 500 spectrometers operating at 27°C. ^1^H-Detected experiments including two-dimensional COSY, NOESY, HSQC, and HMBC were measured with an inverse ^1^H/^13^C probe head; direct ^13^C measurements were performed with a QNP ^13^C/^31^P/^29^Si/^19^F/^1^H cryoprobe. All experiments were done in full automation using standard parameter sets of the TOPSPIN software package (Bruker). ^13^C NMR spectra were recorded in proton-decoupled mode. Data processing was typically done with the MestreNova software.

### Heterologous Gene Expression in *Escherichia coli*

Primers for the amplification of the genes *dch* and *oah* were derived from GenBank sequence no. AJ224959.2. The *dch* gene was expressed with a C-terminal, *oah* with a N-terminal 6x-His tag. PCR parameters for gene amplifications were: 30 sec at 98°C, 30 s at 52°C, and 45 s at 72°C, repeated 10 times before increasing the annealing temperature to 56°C for another 25 cycles; Q5 DNA polymerase (New England Biolabs) was used. The amplicons were ligated with the target vector pOT1, a hybrid of the plasmids pBBR1MCS-2 (Kovach et al., 1995) and pTrc99a (Amann et al., 1988). A fragment comprising *lacIq* and the multiple cloning site of pTrc99a was amplified by PCR and subsequently ligated with a pBBR1 fragment from which the MCS had been removed by *Age*I/*Nsi*I. For primer sequences used see Supplementary Table 1. Gene expressions were carried out in *E. coli* MC4100 cells (Peters et al., 2003) by induction with 1.0 mM isopropyl β-D-1-thiogalactopyranoside (IPTG) at OD_578_ of 0.4 to 0.6, after aerobic growth in LB medium containing 75 μg mL^-1^ kanamycin. After growth overnight at 20°C, the cells were harvested by centrifugation and stored at -20°C until use. Purification of recombinant proteins was carried out in a buffer containing 20 mM TEA/NaOH at pH 7.4, 300 mM NaCl, 20 mM imidazole, 10% glycerol (v/v) and 1 mM DTE. Cell suspensions were passed twice through a French pressure cell at 120 MPa. The cell lysate was centrifuged at 150,000 × *g* for 1 h (4°C) before applying the supernatant to a Ni Sepharose^TM^ High Performance HisTrap^TM^ HP column (GE Healthcare) according to manufacturer’s instructions. After washing with 60 mM imidazole, proteins were eluted by a 120 and 250 mM step gradient. Imidazole was removed afterward using PD MiniTrap G-25 desalting columns (GE Healthcare) according to manufacturer’s instructions. Proteins were stored at -20°C until use.

## Results

### Growth of *T. aromatica* with 2-F-Benzoate and Benzoate

**FIGURE 2:**
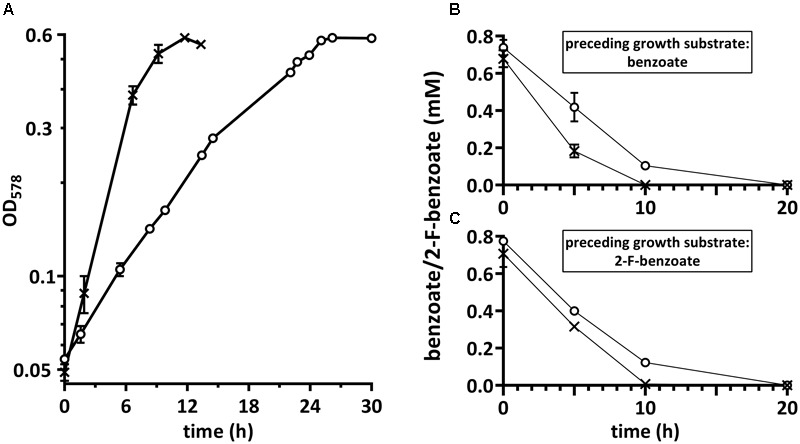
**(A)** Growth of *Thauera aromatica* K172 with 2-F-benzoate (○) and benzoate (×), and the consumption of both of these substrates by suspensions of cells grown with **(B)** benzoate or **(C)** 2-F-benzoate. Both substrates were consumed by either type of cells as indicated by decline of their concentrations. Corresponding data points were connected for comprehensible visualization. The *y*-axes show concentrations of benzoate/2-F-benzoate added to the cell suspensions. Means of two biological replicates are shown.

*Thauera aromatica* was chosen as model organism to study the processes involved in 2-F-benzoate metabolism and to compare them with those of benzoate metabolism in the same organism. Cells were cultivated in a mineral salt medium with 2.7 mM 2-F-benzoate or benzoate as carbon and electron source and 9.5 mM nitrate as electron acceptor (**Figure 2A**). The doubling time averaging 6.4 ± 0.1 h was two to three times higher than with benzoate. When cells reached stationary growth phase, the substrate was completely consumed. With a calculated optical density of 1.0 per 0.22 g L^-1^ of cells (dry weight) the yield was 48 g (dry weight) per mol 2-F-benzoate consumed.

### Whole-Cell Suspension Assays

Cells were tested for the induction of enzymes specifically required for 2-F-benzoate degradation that are not produced during benzoate degradation. For this purpose, consumption of both substrates by dense suspensions (OD_578_ ≈ 8.3) of cells grown with benzoate or 2-F-benzoate was determined. Either type of cells instantly consumed 2-F-benzoate at the rate of 36 ± 4 μmol min^-1^ g^-1^ (dry weight) (**Figures 2B,C**). Consumption of benzoate by cells grown with 2-F-benzoate only slightly decreased to around 70% compared to benzoate-grown cells. In summary, these observations suggested that the enzyme inventory in cells grown with benzoate is adapted to consume 2-F-benzoate readily.

### 2-F-BzCoA Conversion by Cell-Free Extracts

**FIGURE 3:**
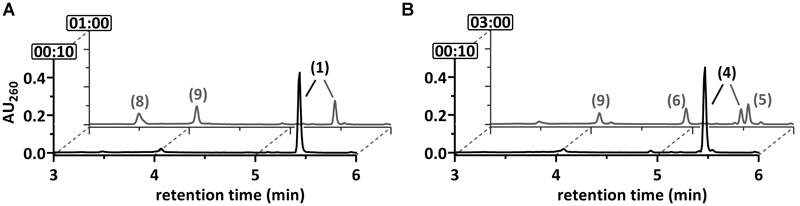
Ultraperformance liquid chromatography (UPLC) diagrams demonstrating the time-dependent *in vitro* conversion of **(A)** 2-F-BzCoA and **(B)** BzCoA by cell-free extracts of *T. aromatica*. UPLC analyses of samples are shown, that were taken at representative time points as indicated by the numbers (min) at the *y*-axis. The peak numbers refer to those assigned to structures in **Figure 1**: 2-F-BzCoA (1), BzCoA (4), 1,5-dienoyl-CoA (5), 6-OH-1-enoyl-CoA (6), 3-OH-pimeloyl-CoA (8), acetyl-CoA (9). Formation of 6-oxo-1-enoyl-CoA was not observed.

Soluble extracts (150,000 × *g* supernatant) of 2-F-benzoate- or benzoate-grown cells both consumed 2-F-BzCoA at a similar rate (80 ± 11 mU mg^-1^, mean value of triplicate determinations ± standard deviation) as observed by ultra-performance liquid chromatography (UPLC) analysis (**Figure 3A**). Similar as during *in vitro* conversion of BzCoA, 2-F-BzCoA conversion strictly depended on Ti(III)-citrate as an artificial electron donor and MgATP. As major products 3-OH-pimeloyl-CoA and acetyl-CoA were identified, that are known as rather late intermediates of the BzCoA degradation pathway (**Figures 1, 3A**). Remarkably, neither fluorinated products nor non-fluorinated intermediates of the upper BzCoA degradation pathway such as 1,5-dienoyl-CoA or its hydrated equivalent 6-OH-1-enoyl-CoA were observed. The latter usually accumulate during BzCoA conversion *in vitro* under the same conditions, when NAD^+^, the electron acceptor of the 3-OH-acyl-CoA dehydrogenase (HAD), was not added to the assay (**Figures 1, 3B**). In conclusion, the conversion of 2-F-BzCoA to 3-OH-pimeloyl-CoA is considered to proceed independently of NAD^+^/HAD.

### 2-F-BzCoA Conversion by Enriched BCR

**FIGURE 4:**
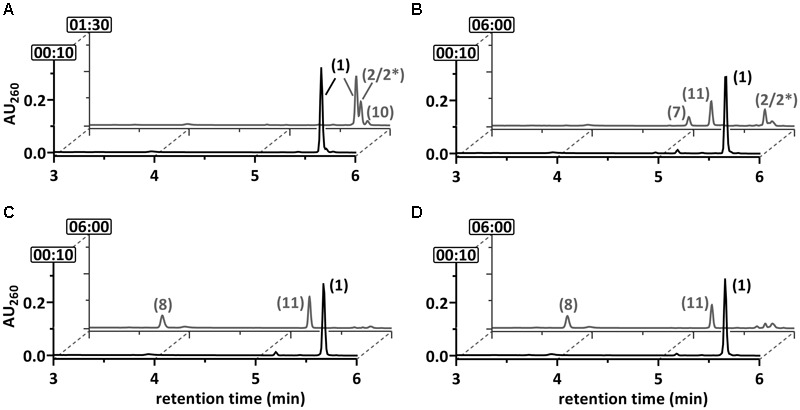
Ultraperformance liquid chromatography diagrams demonstrating the time-dependent conversion of 2-F-BzCoA (1) by **(A)** BCR, **(B)** BCR+DCH, **(C)** BCR+DCH+OAH and **(D)** BCR+OAH. UPLC analyses of samples are shown, that were taken at representative time points as indicated by the numbers (min) at the *y*-axis. The peak numbers refer to those assigned to structures in **Figures 1, 7**: 2-F-BzCoA (1), F-1,5-dienoyl-CoA (2 and/or 2^∗^), 6-oxo-1-enoyl-CoA (7), 3-OH-pimeloyl-CoA (8), F-1-enoyl-CoA (10) and F-OH-1-enoyl-CoA (11).

BzCoA reductase, enriched from cells of *T. aromatica* grown anaerobically with benzoate as described (Boll and Fuchs, 1995), converted 2-F-BzCoA (compound 1) at a rate of 130 mU mg^-1^ to compound 2 and after prolonged incubation to compound 10 (**Figure 4A**). The characteristic UV/vis spectrum of the product strongly resembled that of 1,5-dienoyl-CoA (5) (for UV/vis spectra of CoA esters see Supplementary Figure 1). Mass spectrometric analysis (Q-TOF-MS) identified compound 2 as fluorinated 1,5-dienoyl-CoA (F-1,5-dienoyl-CoA) with an m/z of 892.1564 (theoretical m/z of the [M+H]^+^ ion = 892.1555; for mass spectra of CoA thioesters see Supplementary Figure 3). Depending on the regioselectivity of the BCR-catalyzed reaction two possible isomers of F-1,5-dienoyl-CoA may be formed (2-F-1,5-dienoyl-CoA and 6-F-1,5-dienoyl-CoA; compounds 2 and 2^∗^ in **Figure 7**), that cannot be separated by UPLC. To analyze which of the isomers was formed, ^1^H NMR analysis attempts were carried out. However, during the isolation and NMR measurements the F-1,5-dienoyl-CoA compound(s) readily decomposed to unknown products excluding a NMR based structure analysis. In order to obtain initial insights into the regioselectivity of BCR with fluorinated BzCoA analogs, 3-F-BzCoA was converted by BCR in the presence of Ti(III) citrate and MgATP as the products were expected to be more stable than those of the corresponding 2-F-BzCoA reduction products. ^1^H NMR analyses of the products revealed the formation of both 3-F-1,5-dienoyl-CoA and 5-F-1,5-dienoyl-CoA suggesting a low regioselectivity of BCR for fluorinated BzCoA analogs (Supplementary Figure 2 and Supplementary Table 2). However, a quantitative determination of the 3-F-/5-F-ratio could not be reliably determined due to their decay during prolonged NMR analysis.

Compound 10 was formed only after prolonged incubation, and it was identified by mass spectrometry as a fluorinated cyclohex-1-enoyl-CoA (F-1-enoyl-CoA; measured m/z = 894.1722; theoretical m/z of the [M+H]^+^ ion = 894.1711; Supplementary Figure 3). Its formation can be explained by the artificial reduction of F-1,5-dienoyl-CoA by BCR in the absence of the subsequent enzymes of the BzCoA degradation pathway, which has also been reported during BzCoA/1,5-dienoyl-CoA conversion (Boll et al., 2000). In summary, BCR readily converted 2-F-BzCoA most likely to a 2-F-/6-F-1,5-dienoyl-CoA isomer mixture, whereas no defluorination to BzCoA was observed. This finding suggests that BCR is not involved in C–F-bond cleavage of 2-F-BzCoA as observed for 4-F-BzCoA conversion to BzCoA + HF (Tiedt et al., 2016).

### 2-F-BzCoA Conversion by Enriched BCR and DCH

The results obtained so far suggest that enzymatic defluorination is accomplished by enzymes involved in the conversion of the 2-/6-F-1,5-dienoyl-CoA isomers to 3-OH-pimeloyl-CoA. As cell suspension experiments provided no evidence for a *de novo* synthesis of enzymes, the candidate enzymes involved in C–F-bond cleavage were DCH, HAD, or OAH (**Figure 1**). Taken into account that 2-F-BzCoA conversion to 3-OH-pimeloyl-CoA readily occurred in the absence of NAD^+^, the 3-OH-acyl-CoA dehydrogenase HAD was unlikely to play a role in the defluorination reaction. To test whether the remaining DCH and/or OAH were involved in enzymatic defluorination of 2-/6-F-1,5-dienoyl-CoA intermediates, both enzymes were heterologously produced in *E. coli* with a 6x His-tag followed by high enrichment by Ni-affinity chromatography (Supplementary Figures 4, 5).

Purified recombinant DCH hydrated 1,5-dienoyl-CoA at a rate of 107 ± 12 U mg^-1^ (Supplementary Figure 6A) consistent with formerly reported activity values (110 U mg^-1^ for the reverse reaction) (Laempe et al., 1998). When 2-F-BzCoA was reacted with BCR and DCH, formation of a fluorinated hydroxy-1-enoyl-CoA (F-OH-1-enoyl-CoA, compound 11) was observed (**Figure 4B**), as indicated by mass spectrometric analysis (measured m/z = 910.1666; theoretical m/z of the [M+H]^+^ ion = 910.1660; Supplementary Figure 3). As a further product 6-oxo-1-enoyl-CoA was identified by co-elution with a standard and by mass spectrometric analysis (compound 7, measured m/z = 890.1597; theoretical m/z of the [M+H]^+^ ion = 890.1598; ≈35% peak area compared to F-OH-1-enoyl-CoA). The formation of both, a fluorinated and a non-fluorinated product from 2-F-BzCoA in the presence of BCR and DCH suggests that both F-1,5-dienoyl-CoA isomers formed by BCR were hydrated by DCH, and that hydration of one isomer was accompanied by C–F-bond cleavage. Whereas the 2-F-6-OH-1-enoyl-CoA formed should be considerably stable, the 6-F-6-OH-1-enoyl-CoA represents a highly reactive α-fluorohydrin. Such compounds are prone to spontaneous decomposition to ketones by direct expulsion of fluoride by the adjacent hydroxyl oxygen (Teipel et al., 1968; Seppelt, 1977; Marletta et al., 1982). Hence, the observed formation of 6-oxo-1-enoyl-CoA can be explained by the decay of an unstable 6-F-6-OH-1-enoyl-CoA intermediate. Hence, stable compound 11 is assigned to the 2-F-6-OH-1-enoyl-CoA (**Figures 4B, 7**). The ratio of the two products formed by DCH (compounds 7 and 11) should reflect those of the 2-F- and 6-F-1,5-dienoyl-CoA isomers produced by BCR which is approximately 1:2.

### 2-F-BzCoA Conversion by Enriched BCR, DCH and OAH

In a previous study, the heterologous expression of *oah* from *T. aromatica* in *E. coli* failed (Laempe et al., 1999). Using a modified protocol, we succeeded in heterologous production of OAH with a specific activity of 62.3 ± 8 mU mg^-1^ (Supplementary Figure 5). Though this activity was significantly lower than the activity reported for the enriched wild-type enzyme (Laempe et al., 1999), recombinant OAH was used for *in vitro* defluorination studies.

When 2-F-BzCoA was converted in the presence of BCR, DCH and OAH, again formation of 2-F-6-OH-1-enoyl-CoA was observed. In addition, 3-OH-pimeloyl-CoA (compound 8; measured m/z = 926.1819; theoretical m/z of the [M+H]^+^ ion = 926.1809) instead of 6-oxo-1-enoyl-CoA (compound 7) was identified, which can be interpreted as hydration and hydrolytic ring cleavage of the 6-oxo-1-enoyl-CoA intermediate by OAH (**Figures 1C, 4C**). When DCH was omitted from the assay, surprisingly OAH alone produced a similar 2-F-6-OH-1-enoyl-CoA/3-OH-pimely-CoA mixture (**Figure 4D**). The overall 3-OH-pimeloyl-CoA and 2-F-6-OH-1-enoyl-CoA formation rate in this assay was 19.2 mU mg^-1^, which was approximately threefold lower than the activity measured for the hydrolysis of 6-oxo-1-enoyl-CoA by OAH.

These results indicate that OAH was competent to hydrate both fluorinated 1,5-dienoyl-CoA isomers and exhibited a similar reactivity as DCH. This finding was unexpected as hydration of 1,5-dienoyl-CoA analogs to 6-OH-1-enoyl-CoA products represents a rather unusual 1,4-addition of water, which was considered a unique activity of DCH (Peters et al., 2007). Remarkably, OAH only hydrated the fluorinated dienoyl-CoA isomers formed from 2-F-BzCoA by BCR, whereas conversion of non-fluorinated 1,5-dienoyl-CoA was negligible (Supplementary Figure 6). In conclusion, DCH and OAH can both initiate C–F bond cleavage by hydration of the 2-F-1,5-dienoyl-CoA isomer to an unstable α-fluorohydrin (**Figure 7**). The product of spontaneous decomposition of the latter yields 6-oxo-1-enoyl-CoA that is readily hydrolyzed to 3-OH-pimeloyl-CoA by OAH but not by DCH.

### 2-F-6-OH-1-Enoyl-CoA Conversion by DCH and OAH

**FIGURE 5:**
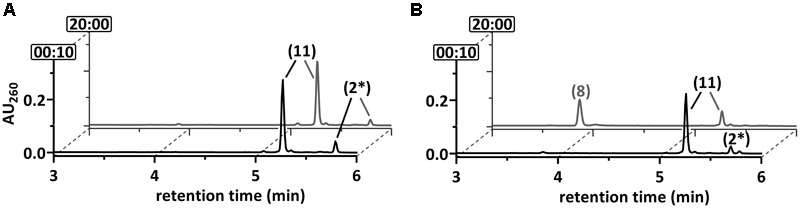
Ultraperformance liquid chromatography diagrams demonstrating the time-dependent conversion of 2-F-6-OH-1-enoyl-CoA. The diagrams show the time-dependent conversion by **(A)** DCH or **(B)** DCH+OAH. UPLC analyses of samples are shown, that were taken at representative time points as indicated by the numbers (min) at the *y*-axis. The peak numbers refer to those assigned to structures in **Figures 1, 7**: 6-F-1,5-dienoyl-CoA (2^∗^), 3-OH-pimeloyl-CoA (8), 2-F-6-OH-monoenoyl-CoA (11).

The results obtained so far clearly suggested that DCH and OAH both initiate defluorination of the 2-F-1,5-dienoyl-CoA intermediate by 1,4-hydration. However, it was unclear which of the two enzymes was involved in defluorination of the stable 2-F-6-OH-1-enoyl-CoA. For this reason, the latter was enzymatically synthesized from 2-F-BzCoA using BCR/DCH and isolated by HPLC at the mg scale; it was stable for hours at room temperature. Upon reaction with DCH, 2-F-6-OH-1-enoyl-CoA was dehydrated to 6-F-1,5-dienoyl-CoA (79 U mg^-1^), representing the reverse reaction of DCH with an equilibrium far on the side of the 2-F-6-OH-1-enoyl-CoA (**Figure 5A**). However, no conversion to 6-oxo-1-enoyl-CoA was observed, confirming that only the 2-F- but not the 6-F-1,5-dienoyl-CoA can be defluorinated by DCH. Addition of OAH resulted in the immediate formation of 3-OH-pimeloyl-CoA (5.0 mU mg^-1^) (**Figure 5B**) indicating that OAH initiated C–F-bond cleavage of 2-F-6-OH-1-enoyl-CoA. The most likely scenario is that OAH catalyzed a 1,2-addition of water resulting in the formation of the unstable α-fluorohydrin 2-F-2,6-di-OH-cyclohexanoyl-CoA that should readily decompose to HF and 2-oxo-6-OH-cyclohexanoyl-CoA (compound 12). The latter is the likely water-adduct intermediate during the conversion of 6-oxo-1-enoyl-CoA to 3-OH-pimeloyl-CoA by OAH. In conclusion, OAH alone is capable of defluorinating both F-1,5-dienoyl-CoA isomers formed by BCR (**Figure 7**).

### Growth of Selected Bacterial Strains with 2-F-Benzoate

The results obtained suggest that class I BCR generates the 2-F- and 6-F-1,5-dienoyl-CoA isomers, which both can be defluorinated by the action of OAH (and DCH in case of the 2-F-1,5-dienoyl-CoA). OAH is highly conserved in all bacteria capable of anaerobically degrading benzoate and other monocyclic aromatic compounds via the BzCoA degradation pathway (Kuntze et al., 2008). Assuming that the ATP-independent class II BCR, present in strictly anaerobic bacteria, generates the same 2-F-/6-F-1,5-dienoyl-CoA products as the class I enzyme, 2-F-benzoate should serve as growth substrate for all facultatively and strictly anaerobic bacteria that degrade monocyclic aromatic compounds via the BzCoA degradation pathway.

To prove this hypothesis, we tested several anaerobic benzoate-degrading strains for the additional capability of growing with 2-F-benzoate as only source of carbon and energy in the presence of appropriate electron acceptors (**Table 1**). All denitrifying bacteria tested of the genera *Thauera, Azoarcus*, and ‘*Aromatoleum’* grew with 2-F-benzoate at doubling times approximately two to three times higher than with benzoate. Moreover, we observed previously unknown growth of aromatic compound degrading, sulfate reducing bacteria including Gram-positives (*Desulfotomaculum gibsoniae*) and δ-Proteobacteria (*Desulfobacterium anilini, Desulfococcus multivorans*). Here, the growth rates were only slightly lower than with benzoate, presumably because sulfate reduction rather than BzCoA degradation was rate-limiting in the overall energy metabolism. However, there were also exceptions: even after several trials, no growth was observed for the iron-reducing strain *Geobacter metallireducens* GS-15 and the sulfate reducing strain *Desulfosarcina cetonica*.

**Table 1:** Anaerobic growth of selected bacterial strains with 2-F-benzoate.

	Doubling time (h)	
	2-F-benzoate	Benzoate
*Thauera aromatica* K172	6.4	2.2
*Thauera chlorobenzoica* 3CB-1^∗^	14	5.0
*Azoarcus* sp. CIB	16	5.4
*Azoarcus evansii* KB740^∗^	14	4.3
*Aromatoleum aromaticum* EbN1	6.3	3.8
*Desulfobacterium anilini*	130	56
*Desulfococcus multivorans*	9.0	8.1
*Desulfotomaculum gibsoniae*	144	112
*Desulfosarcina cetonica*	–	27
*Geobacter metallireducens* GS-15	*–*	6.2

*^∗^Strains were reported for anaerobic growth with 2-F-benzoate before (Schennen et al., 1985; Anders et al., 1995).*

### 2-Cl-BzCoA Conversion by Enriched BCR, DCH and OAH

**FIGURE 6:**
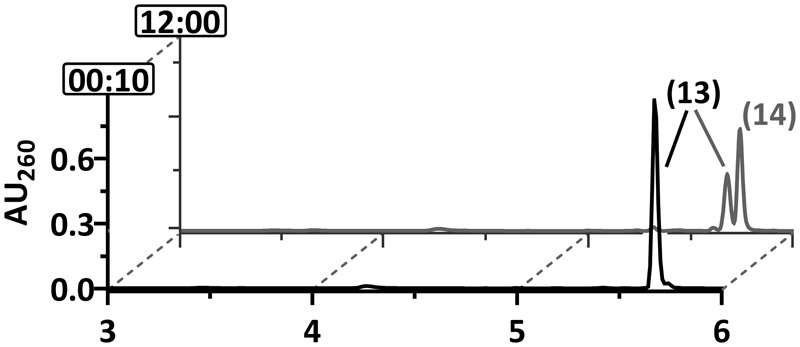
Ultraperformance liquid chromatography diagrams demonstrating the time-dependent conversion of 2-Cl-BzCoA by BCR+DCH and OAH. UPLC analyses of samples are shown, that were taken at representative time points as indicated by the numbers (min) at the *y*-axis. Peaks of CoA ester compounds are numbered as follows: 2-Cl-BzCoA (13), Cl-1,5-dienoyl-CoA (14).

None of the strains capable of degrading 2-F-benzoate was capable of growing with 2-Cl-benzoate. We therefore tested whether dehalogenation in *ortho*-position is feasible when chlorine substitutes for fluorine. In accordance with earlier reports (Möbitz and Boll, 2002), BCR ATP- and Ti(III) citrate-dependently converted 2-Cl-BzCoA to chlorinated 1,5-dienoyl-CoA (measured m/z = 906,1106; theoretical m/z of the [M+H]^+^ ion = 906,1103). However, in contrast to the F-1,5-dienoyl-CoA isomers, the chlorinated analog was neither converted by DCH nor by OAH (**Figure 6**). This finding explains why no growth with 2-Cl-benzoate was observed.

## Discussion

**FIGURE 7:**
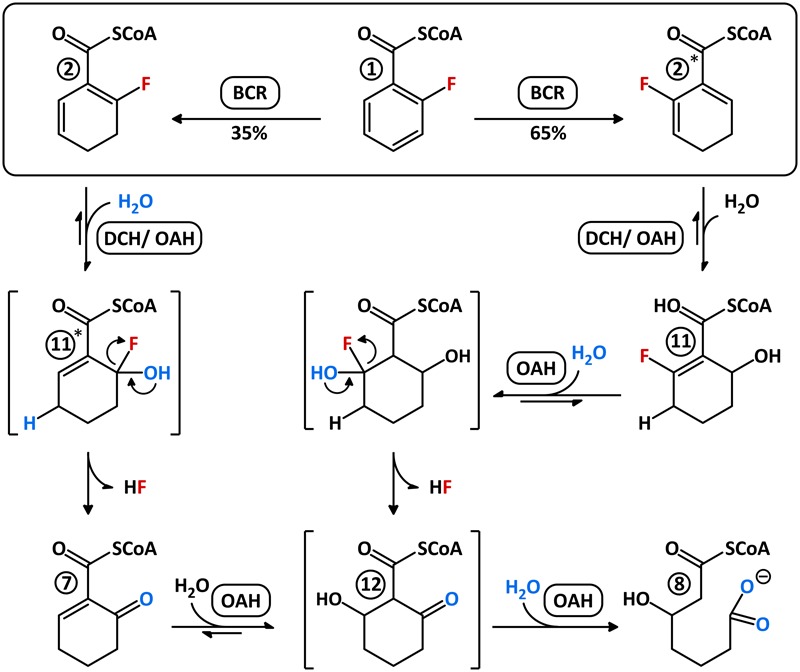
Possible mechanism for the conversion of 2-F-BzCoA by BCR, DCH, and OAH. Both F-1,5-dienoyl-CoA isomers (compounds 2/2^∗^) are hydrated to different F-OH-1-enoyl-CoA isomers (compounds 11/11^∗^) by DCH and OAH, likewise. Unstable 6-F-6-OH-1-enoyl-CoA (11^∗^) spontaneously decomposes to 6-oxo-1-enoyl-CoA (compound 7) by HF-expulsion. This, in the presence of OAH, becomes immediately hydrated presumably to 2-oxo-6-OH-cyclohexanoyl-CoA (compound 12) before hydrolysis to 3-OH-pimeloyl-CoA (compound 8). Stable 2-F-6-OH-1-enoyl-CoA (compound 11) can also only be further hydrated by OAH, apparently to the unstable 2-F-2,6-di-OH-cyclohexanoyl-CoA intermediate, which spontaneously decomposes to compound 12 before ring hydrolysis by OAH. Intermediates illustrated with brackets probably only occur transiently.

Using numerous *in vitro* assays with extracts of *T. aromatica* cells grown with 2-F-benzoate and with purified recombinant enzymes, we unraveled the previously unknown metabolites and enzymes involved in anaerobic 2-F-benzoate degradation. The individual enzymatic steps can be summarized as follows (**Figure 7**): (i) activation of 2-F-benzoate to 2-F-BzCoA by promiscuous BCL; (ii) reduction of 2-F-BzCoA to a 1:2 mixture of 2-F-/6-F-1,5-dienoyl-CoA (compounds 2/2^∗^) by promiscuous ATP-dependent class I BCR; (iii) 1,4-hydration of 2-F-1,5-dienoyl-CoA isomer by either DCH or OAH to an instable 6-F-6-OH-1-enoyl-CoA (compound 11^∗^) that spontaneously decomposes to HF and 6-oxo-1-enoyl-CoA, and 1,4-hydration of the 6-F-1,5-dienoyl-CoA to stable 2-F-6-OH-1-enoyl-CoA (compound 11); (iv) conversion of 2-F-6-OH-1-enoyl-CoA to the unstable 2-F-2,6-di-OH-cyclohexane-carboxyl-CoA via 1,2-hydration, catalyzed by OAH. The latter spontaneously decomposes to 2-oxo-6-OH-cyclohexane-carboxyl-CoA (compound 12), which is readily hydrolyzed to 3-OH-pimeloyl-CoA by OAH (**Figure 7**). Similar to 4-F-benzoate degradation, catabolism of 2-F-benzoate does not require any additional enzyme for removal of the substituent at the aromatic ring, which is different to the degradation of 3-chloro-, hydroxy-, or methylbenzoates (Laempe et al., 2001; Kuntze et al., 2011; Lahme et al., 2012; Juárez et al., 2013).

Unlike 4-F-benzoate degradation, where class I BCR is directly involved in reductive defluorination, the downstream enzymes OAH and DCH are employed for C–F-bond cleavage during 2-F-benzoate degradation. As OAH alone is capable of catalyzing the defluorination of both F-1,5-dienoyl-CoA isomers formed by BCR to 3-OH-pimeloyl-CoA and HF, 2-F-benzoate degradation appears to be rather independent of DCH. Moreover, the NAD^+^-dependent HAD is dispensable as F-1,5-dienoyl-CoA and 3-OH-pimeloyl-CoA already have the same oxidation state. As all anaerobic bacteria contain similar BCLs, BCRs, and OAHs (Kuntze et al., 2008), 2-F-benzoate was predicted to serve as growth substrate for all anaerobic bacteria employing the BzCoA degradation pathway. Indeed, we newly identified growth with a number of sulfate-reducing and nitrate-reducing bacteria with 2-F-benzoate. The observed lower growth rate vs. benzoate in denitrifying bacteria can be rationalized by the slow conversion of the 2-F-6-OH-1-enoyl-CoA intermediate by OAH, making this reaction probably the overall rate-limiting step of 2-F-benzoate degradation. With *G. metallireducens* and *D. cetonica* also two exceptions were identified. The inability of growth with 2-F-benzoate might be explained by BCR and/or OAH variants that non-sufficiently convert the fluorinated substrate analogs. Another possibility is that these strains are particularly sensitive to toxic fluoride released during 2-F-benzoate degradation, e.g., due to an insufficient fluoride export (Baker et al., 2012).

Growth with 2-Cl-benzoate and 3-F-benzoate was not observed with any of the tested strains. Although chlorine is even a better leaving group than fluorine, steric effects might be responsible for the inability of OAH and DCH to convert the corresponding Cl-1,5-dienoyl-CoA analogs. In the case of 3-F-BzCoA degradation, no α-fluorohydrins can be formed by DCH or OAH. Degradation of 3-F-BzCoA by promiscuous enzymes of the BzCoA degradation pathway would eventually result in the formation of toxic fluoroacetyl-CoA, which was then metabolized to fluorocitrate (Peters et al., 1953), a potent inhibitor of aconitase (Lauble et al., 1996) and of citrate transport across the mitochondrial membrane (Kun et al., 1977).

The defluorination activities of the crotonase-family members OAH and DCH have not been reported before, and both are considered to proceed via unstable α-fluorohydrin intermediates. The hydration of 2-F-1,5-dienoyl-CoA (compound 2) to 6-oxo-1-enoyl-CoA (compound 7) by OAH represents a formal 1,4-hydration. This activity is surprising because non-fluorinated 1,5-dienoyl-CoA is not a substrate of the enzyme. As OAH catalyzes two different water additions to a 1-enoyl-CoA (1,2-addition) and a ketone moiety (hydrolytic ring cleavage), OAH exhibits a catalytic flexibility that might be essential for defluorination of both, 2-F-1,5-dienoyl-CoA and 2-F-6-OH-1-enoyl-CoA (Laempe et al., 1999; Kuntze et al., 2008). In both fluorinated substrates, the fluorine substituent is bonded to a sp^2^-hybridized carbon that alters the electronic properties of the molecule by its +M- and -I-effect. As a result, both mimic the electronic properties of 6-oxo-1-enoyl-CoA/2-oxo-1-OH-cyclohexanoyl-CoA, the natural substrate/reaction intermediate of OAH.

A similar mode of defluorination has been reported for fumarase that converts 2-F-fumarate to oxaloacetate and HF, and an α-fluorohydrin intermediate has also been proposed for this reaction (Teipel et al., 1968; Marletta et al., 1982). While 2-F-fumarate defluorination is physiologically irrelevant, defluorination by OAH/DCH is essential for using 2-F-benzoate as carbon and energy source. It is tempting to speculate whether the defluorination activities identified in this work are also relevant during complete degradation of other fluoroaromatics that are likely to be degraded via 2-F-BzCoA, e.g., 2-F-toluene, F-benzene, 3-F-phenol (presumably carboxylated to 2-F-4-OH-benzoate) and many others.

## Author Contributions

OT carried out the experimental work. OT and MB designed the experiments. MM contributed to mass spectrometric measurements and the analyses of the acquired data. WE performed NMR spectroscopic experiments and analyzed the data. OT and MB wrote and critically revised the manuscript.

## Conflict of Interest Statement

The authors declare that the research was conducted in the absence of any commercial or financial relationships that could be construed as a potential conflict of interest.

## References

[B1] Altenschmidt U. Oswald B. Fuchs G. (1991). Purification and characterization of benzoate-coenzyme A ligase and 2-aminobenzoate-coenzyme A ligases from a denitrifying *Pseudomonas* sp. *J. Bacteriol.* 173 5494–5501. 10.1128/jb.173.17.5494-5501.1991 1885526 PMC208262

[B2] Amann E. Ochs B. Abel K.-J. (1988). Tightly regulated tac promoter vectors useful for the expression of unfused and fused proteins in *Escherichia coli*. *Gene* 69 301–315. 10.1016/0378-1119(88)90440-4 3069586

[B3] Anders H. J. Kaetzke A. Kämpfer P. Ludwig W. Fuchs G. (1995). Taxonomic position of aromatic-degrading denitrifying pseudomonad strains K 172 and KB 740 and their description as new members of the genera *Thauera*, as *Thauera aromatica* sp. nov., and *Azoarcus*, as *Azoarcus evansii* sp. nov., respectively, members of the beta subclass of the Proteobacteria. *Int. J. Syst. Bacteriol.* 45 327–333. 10.1099/00207713-45-2-327 7537067

[B4] Baker J. L. Sudarsan N. Weinberg Z. Roth A. Stockbridge R. B. Breaker R. R. (2012). Widespread genetic switches and toxicity resistance proteins for fluoride. *Science* 335 233–235. 10.1126/science.1215063 22194412 PMC4140402

[B5] Boll M. Fuchs G. (1995). Benzoyl-coenzyme a reductase (dearomatizing), a key enzyme of anaerobic aromatic metabolism. ATP dependence of the reaction, purification and some properties of the enzyme from *Thauera aromatica* strain K172. *Eur. J. Biochem.* 234 921–933. 10.1111/j.1432-1033.1995.921_a.x 8575453

[B6] Boll M. Laempe D. Eisenreich W. Bacher A. Mittelberger T. Heinze J. (2000). Nonaromatic products from anoxic conversion of benzoyl-CoA with benzoyl-CoA reductase and cyclohexa-1,5-diene-1-carbonyl-CoA hydratase. *J. Biol. Chem.* 275 21889–21895. 10.1074/jbc.M001833200 10766750

[B7] Cooper M. Wagner A. Wondrousch D. Sonntag F. Sonnabend A. Brehm M. (2015). Anaerobic microbial transformation of halogenated aromatics and fate prediction using electron density modeling. *Environ. Sci. Technol.* 49 6018–6028. 10.1021/acs.est.5b00303 25909816

[B8] Egland P. G. Gibson J. Harwood C. S. (2001). Reductive, coenzyme A-mediated pathway for 3-chlorobenzoate degradation in the phototrophic bacterium *Rhodopseudomonas palustris*. *Appl. Environ. Microbiol.* 67 1396–1399. 10.1128/AEM.67.3.1396-1399.2001 11229940 PMC92743

[B9] Erb T. J. Berg I. A. Brecht V. Müller M. Fuchs G. Alber B. E. (2007). Synthesis of C5-dicarboxylic acids from C2-units involving crotonyl-CoA carboxylase/reductase: the ethylmalonyl-CoA pathway. *Proc. Natl. Acad. Sci. U.S.A.* 104 10631–10636. 10.1073/pnas.0702791104 17548827 PMC1965564

[B10] Holliger C. Schumacher W. (1994). Reductive dehalogenation as a respiratory process. *Antonie Van Leeuwenhoek* 66 239–246. 10.1007/BF00871642 7747935

[B11] Hug L. A. Maphosa F. Leys D. Löffler F. E. Smidt H. Edwards E. A. (2013). Overview of organohalide-respiring bacteria and a proposal for a classification system for reductive dehalogenases. *Philos. Trans. R. Soc. B Biol. Sci.* 368:20120322. 10.1098/rstb.2012.0322 23479752 PMC3638463

[B12] Juárez J. F. Zamarro M. T. Eberlein C. Boll M. Carmona M. Díaz E. (2013). Characterization of the mbd cluster encoding the anaerobic 3-methylbenzoyl-CoA central pathway. *Environ. Microbiol.* 15 148–166. 10.1111/j.1462-2920.2012.02818.x 22759228

[B13] Jugder B.-E. Ertan H. Bohl S. Lee M. Marquis C. P. Manefield M. (2016). Organohalide respiring bacteria and reductive dehalogenases: key tools in organohalide bioremediation. *Front. Microbiol.* 7:209. 10.3389/fmicb.2016.00249 26973626 PMC4771760

[B14] Key B. D. Howell R. D. Criddle C. S. (1997). Fluorinated organics in the biosphere. *Environ. Sci. Technol.* 31 2445–2454. 10.1021/es961007c

[B15] Kiel M. Engesser K.-H. (2015). The biodegradation vs. biotransformation of fluorosubstituted aromatics. *Appl. Microbiol. Biotechnol.* 99 7433–7464. 10.1007/s00253-015-6817-5 26216240

[B16] Kovach M. E. Elzer P. H. Steven Hill D. Robertson G. T. Farris M. A. Roop R. M. (1995). Four new derivatives of the broad-host-range cloning vector pBBR1MCS, carrying different antibiotic-resistance cassettes. *Gene* 166 175–176. 10.1016/0378-1119(95)00584-1 8529885

[B17] Kun E. Kirsten E. Sharma M. L. (1977). Enzymatic formation of glutathione-citryl thioester by a mitochondrial system and its inhibition by (-) erythrofluorocitrate. *Proc. Natl. Acad. Sci. U.S.A.* 74 4942–4946. 270728 10.1073/pnas.74.11.4942 PMC432073

[B18] Kuntze K. Kiefer P. Baumann S. Seifert J. Bergen M. Vorholt J. A. (2011). Enzymes involved in the anaerobic degradation of *meta*-substituted halobenzoates. *Mol. Microbiol.* 82 758–769. 10.1111/j.1365-2958.2011.07856.x 22010634

[B19] Kuntze K. Shinoda Y. Moutakki H. Mcinerney M. J. Vogt C. Richnow H.-H. (2008). 6-Oxocyclohex-1-ene-1-carbonyl-coenzyme A hydrolases from obligately anaerobic bacteria: characterization and identification of its gene as a functional marker for aromatic compounds degrading anaerobes. *Environ. Microbiol.* 10 1547–1556. 10.1111/j.1462-2920.2008.01570.x 18312395

[B20] Laempe D. Eisenreich W. Bacher A. Fuchs G. (1998). Cyclohexa-1,5-diene-1-carboxyl-CoA hydratase, an enzyme involved in anaerobic metabolism of benzoyl-CoA in the denitrifying bacterium *Thauera aromatica*. *Eur. J. Biochem.* 255 618–627. 10.1046/j.1432-1327.1998.2550618.x 9738901

[B21] Laempe D. Jahn M. Breese K. Schägger H. Fuchs G. (2001). Anaerobic metabolism of 3-hydroxybenzoate by the denitrifying bacterium *Thauera aromatica*. *J. Bacteriol.* 183 968–979. 10.1128/JB.183.3.968-979.2001 11208796 PMC94965

[B22] Laempe D. Jahn M. Fuchs G. (1999). 6-Hydroxycyclohex-1-ene-1-carbonyl-CoA dehydrogenase and 6-oxocyclohex-1-ene-1-carbonyl-CoA hydrolase, enzymes of the benzoyl-CoA pathway of anaerobic aromatic metabolism in the denitrifying bacterium *Thauera aromatica*. *Eur. J. Biochem.* 263 420–429. 10.1046/j.1432-1327.1999.00504.x 10406950

[B23] Lahme S. Eberlein C. Jarling R. Kube M. Boll M. Wilkes H. (2012). Anaerobic degradation of 4-methylbenzoate via a specific 4-methylbenzoyl-CoA pathway. *Environ. Microbiol.* 14 1118–1132. 10.1111/j.1462-2920.2011.02693.x 22264224

[B24] Lauble H. Kennedy M. C. Emptage M. H. Beinert H. Stout C. D. (1996). The reaction of fluorocitrate with aconitase and the crystal structure of the enzyme-inhibitor complex. *Proc. Natl. Acad. Sci. U.S.A.* 93 13699–13703. 8942997 10.1073/pnas.93.24.13699 PMC19395

[B25] Marletta M. A. Cheung Y.-F. Walsh C. (1982). Stereochemical studies on the hydration of monofluorofumarate and 2,3-difluorofumarate by fumarase. *Biochemistry* 21 2637–2644. 10.1021/bi00540a010 7093213

[B26] Mechichi T. Stackebrandt E. Gad’on N. Fuchs G. (2002). Phylogenetic and metabolic diversity of bacteria degrading aromatic compounds under denitrifying conditions, and description of *Thauera phenylacetica* sp. nov., *Thauera aminoaromatica* sp. nov., and *Azoarcus buckelii* sp. nov. *Arch. Microbiol.* 178 26–35. 10.1007/s00203-002-0422-6 12070766

[B27] Möbitz H. Boll M. (2002). A birch-like mechanism in enzymatic benzoyl-CoA reduction: a kinetic study of substrate analogues combined with an ab initio model. *Biochemistry* 41 1752–1758. 10.1021/bi0113770 11827519

[B28] Murphy C. D. (2010). Biodegradation and biotransformation of organofluorine compounds. *Biotechnol. Lett.* 32 351–359. 10.1007/s10529-009-0174-3 19943179

[B29] Murphy C. D. Clark B. R. Amadio J. (2009). Metabolism of fluoroorganic compounds in microorganisms: impacts for the environment and the production of fine chemicals. *Appl. Microbiol. Biotechnol.* 84 617–629. 10.1007/s00253-009-2127-0 19629474

[B30] Murphy C. D. Quirke S. Balogun O. (2008). Degradation of fluorobiphenyl by *Pseudomonas pseudoalcaligenes* KF707. *FEMS Microbiol. Lett.* 286 45–49. 10.1111/j.1574-6968.2008.01243.x 18616594

[B31] Natarajan R. Azerad R. Badet B. Copin E. (2005). Microbial cleavage of CF bond. *J. Fluor. Chem.* 126 424–435. 10.1016/j.jfluchem.2004.12.001 25114233

[B32] Park B. K. Kitteringham N. R. O’Neill P. M. (2001). Metabolism of fluorine-containing drugs. *Annu. Rev. Pharmacol. Toxicol.* 41 443–470. 10.1146/annurev.pharmtox.41.1.443 11264465

[B33] Peters F. Rother M. Boll M. (2004). Selenocysteine-containing proteins in anaerobic benzoate metabolism of *Desulfococcus multivorans*. *J. Bacteriol.* 186 2156–2163. 10.1128/JB.186.7.2156-2163.2004 15028701 PMC374395

[B34] Peters F. Shinoda Y. Mcinerney M. J. Boll M. (2007). Cyclohexa-1,5-diene-1-carbonyl-coenzyme A (CoA) hydratases of *Geobacter metallireducens* and *Syntrophus aciditrophicus*: evidence for a common benzoyl-CoA degradation pathway in facultative and strict anaerobes. *J. Bacteriol.* 189 1055–1060. 10.1128/JB.01467-06 17122342 PMC1797300

[B35] Peters J. E. Thate T. E. Craig N. L. (2003). Definition of the *Escherichia coli* MC4100 genome by use of a DNA array. *J. Bacteriol.* 185 2017–2021. 10.1128/JB.185.6.2017-2021.2003 12618467 PMC150127

[B36] Peters R. Wakelin R. W. Buffa P. (1953). Biochemistry of fluoroacetate poisoning the isolation and some properties of the fluorotricarboxylic acid inhibitor of citrate metabolism. *Proc. R. Soc. Lond. B Biol. Sci.* 140 497–506. 10.1098/rspb.1953.0004 13027279

[B37] Pfennig N. (1978). *Rhodocyclus purpureus* gen. nov. and sp. nov., a ring-shaped, vitamin B12-requiring member of the family *Rhodospirillaceae*. *Int. J. Syst. Evol. Microbiol.* 28 283–288. 10.1099/00207713-28-2-283

[B38] Purser S. Moore P. R. Swallow S. Gouverneur V. (2008). Fluorine in medicinal chemistry. *Chem. Soc. Rev.* 37 320–330. 10.1039/b610213c 18197348

[B39] Schachter D. Taggart J. V. (1953). Benzoyl-CoA and hippurate synthesis. *J. Biol. Chem.* 203 925–934. 13084662

[B40] Schennen U. Braun K. Knackmuss H. J. (1985). Anaerobic degradation of 2-fluorobenzoate by benzoate-degrading, denitrifying bacteria. *J. Bacteriol.* 161 321–325. 2857161 10.1128/jb.161.1.321-325.1985 PMC214874

[B41] Seppelt K. (1977). Trifluoromethanol, CF3OH. *Angew. Chem. Int. Ed. Engl.* 16 322–323. 10.1002/anie.197703221

[B42] Smart B. E. (2001). Fluorine substituent effects (on bioactivity). *J. Fluor. Chem.* 109 3–11. 10.1016/S0022-1139(01)00375-X

[B43] Song B. Palleroni N. J. Häggblom M. M. (2000). Isolation and characterization of diverse halobenzoate-degrading denitrifying bacteria from soils and sediments. *Appl. Environ. Microbiol.* 66 3446–3453. 10.1128/AEM.66.8.3446-3453.2000 10919805 PMC92169

[B44] Teipel J. W. Hass G. M. Hill R. L. (1968). The substrate specificity of fumarase. *J. Biol. Chem.* 243 5684–5694. 5748979

[B45] Thiele B. Rieder O. Golding B. T. Müller M. Boll M. (2008a). Mechanism of enzymatic birch reduction: stereochemical course and exchange reactions of benzoyl-CoA reductase. *J. Am. Chem. Soc.* 130 14050–14051. 10.1021/ja805091w 18826310

[B46] Thiele B. Rieder O. Jehmlich N. Bergen M. Müller M. Boll M. (2008b). Aromatizing cyclohexa-1,5-diene-1-carbonyl-coenzyme A oxidase. *J. Biol. Chem.* 283 20713–20721. 10.1074/jbc.M802841200 18505724 PMC3258955

[B47] Tiedt O. Mergelsberg M. Boll K. Müller M. Adrian L. Jehmlich N. (2016). ATP-dependent C–F bond cleavage allows the complete degradation of 4-fluoroaromatics without oxygen. *mBio* 7:e00990-16. 10.1128/mBio.00990-16 27507824 PMC4992971

[B48] Tschech A. Pfennig N. (1984). Growth yield increase linked to caffeate reduction in *Acetobacterium woodii*. *Arch. Microbiol.* 137 163–167. 10.1007/bf00414460

[B49] Weinstein L. H. Davison A. (2005). *Fluorides in the Environment: Effects on Plants and Animals.* Wallingford: CABI.

[B50] Wischgoll S. Heintz D. Peters F. Erxleben A. Sarnighausen E. Reski R. (2005). Gene clusters involved in anaerobic benzoate degradation of *Geobacter metallireducens*. *Mol. Microbiol.* 58 1238–1252. 10.1111/j.1365-2958.2005.04909.x 16313613

[B51] Zhang X.-J. Lai T.-B. Kong R. Y.-C. (2012). “Biology of fluoro-organic compounds,” in *Fluorous Chemistry* ed. Horváth I. T. (Berlin: Springer) 365–404. 10.1007/128_2011_270 21952847

