# Supplementary material for: Promiscuous Defluorinating Enoyl-CoA Hydratases/Hydrolases Allow for Complete Anaerobic Degradation of 2-Fluorobenzoate
Source: Front Microbiol. 2017 Dec 21;8:2579. doi: 10.3389/fmicb.2017.02579 (PMC5742626; doi:10.3389/fmicb.2017.02579)
Supplement: Supplementary file 1 [file Data_Sheet_1.pdf]

Promiscuous defluorinating enoyl-CoA hydratases/hydrolases allow for complete anaerobic degradation of 2-fluorobenzoate

Oliver Tiedt[1], Mario Mergelsberg[1], Wolfgang Eisenreich[2], Matthias Boll[1]*

[1]Faculty of Biology – Microbiology, University of Freiburg, Germany

[2]Lehrstuhl für Biochemie, Technische Universität München, Germany

---

Supplementary data

Supplementary Table 1. Primer sequences used to amplify *dch* and *oah* genes from *T. aromatica* or the DNA-region of the plasmid pTrc99a comprising the multiple cloning site (MCS) and *lacI*. Sequences are depicted from 5'- to 3'-ends. Bold segments represent the regions that are homolog to the corresponding ends of the genes or DNA-regions to be amplified. The reverse primer for *dch* and the forward primer for *oah* contain extensions each encoding an N- or C-terminal His-tag, respectively. Start and stop codons (lower-case characters) are displaced correspondingly.

gene/ DNA-region	sequence direction	primer sequence
dch	forward	CATGAATTC**ATGAGCGAGGCCAGCTC**
	reverse	CATAAGCTtcaGTGATGGTGATGGTGATGACCTGACGA**ACGGTTCTCCCAGTTTGC**
oah	forward	CATGGATCCatgCATCACCATCACCATCACGGTTCGTCA**ATGAATCCGACTACGCAAAAAC**
	reverse	CATAAGCTT**CTCCAGCCAGACCTTCAG**
MCS/ *lacI*	forward	TCAACCGGT**CCATCGAATGGTGCAAAACC**
	reverse	TCAATGCAT**CAAAAAGAGTTTGTAGAAACGC**

Supplementary Table 2. Chemical shifts of expected products of 3-F-BzCoA after reduction by class I BCR. Positions refer to chemical structures shown by Supplementary Figure 2. The shift values are referenced to external deuterium oxide. Signal multiplicities are indicated in parentheses (s, singlet; d, doublet; t, triplet; m, multiplet; dd, double doublet; td, triplet of doublets; db, doublets of broads; ddd, doublet of doublet of doublets). n.d.: not determined. F-1,5-dienoyl-CoA isomers were distinguished by means of characteristic signals with COSY correlation. These shift values were close to theoretical values. 3-F-1,5-dienoyl-CoA was identified by a discriminating signal at 5.62 ppm coupling to a signal at 5.93 ppm in HH-COSY. This coupling was considered characteristic for the expected double bond at C5-6. The signal at 6.90 ppm was distinctive for the proton at C2 in structure **(A)** (Supplementary Figure 2). 5-F-1,5-dienoyl-CoA could be distinguished by a triplet signal at 6.69 ppm (H-6) coupled to H-5 at 2.56 ppm. For an additional doublet signal at 5.75 ppm (H-2) only a weak coupling to distant H-4 at 2.40 ppm was observed. Thus, the doublet signal was rather due to coupling to the closer fluorine at C3.

position	chemical shifts (ppm)			
	3-F-1,5-dienoyl-CoA		5-F-1,5-dienoyl-CoA	
	estimated	experimental	estimated	experimental
1	-	-	-	-
2	**6.71**	**6.90 (db) 1H**	**6.71**	**6.69 (t) 1H**
3	3.98	3.38 (m)	**2.15**	**2.56 (m) 2H**
4	2.19	1.66 (m) 1H	**2.15**	**2.40 (t) 2H**
4"	2.44	1.82 (m)	-	-
5	**5.80**	**5.62 (db) 1H**	-	-
6	**5.90**	**5.93 (m) 1H**	**5.50**	**5.75 (d) 1H**
e1	3.13	3.14 (t) 2H	3.13	3.14 (t) 2H
e2	3.87	3.41 (t) 2H	3.87	3.41 (t) 2H
NH-e3	8.01	-	8.01	-
p1	-	-	-	-
p2	2.66	2.43 (t) 2H	2.66	2.43 (t) 2H
p3	3.48	3.44 (t) 2H	3.48	3.44 (t) 2H
NH-p4	8.01	-	8.01	-
p5	-	-	-	-
p6	4.24	4.02 (s) 1H	4.24	4.02 (s) 1H
p7	4.02	-	4.02	-
p8	0.89	0.77 (s) 3H	0.89	0.77 (s) 3H
p9	0.89	0.91 (s) 3H	0.89	0.91 (s) 3H
p10	4.02	3.85 (dd) 1H	4.02	3.85 (dd) 1H
p10'	3.77	3.56 (dd) 1H	3.77	3.56 (dd) 1H
1'	6.16	6.07 (d) 1H	6.16	6.07 (d) 1H
2'	4.75	4.80 (n.d.)	4.75	4.80 (n.d.) n.d.
3'	3.85	n.d.	3.85	n.d.
4'	4.40	4.57 (s) 1H	4.40	4.57 (s) 1H
5'	4.28	4.25 (s) 2H	4.28	4.25 (s) 2H
a2	8.58	8.11 (s) 1H	8.58	8.11 (s) 1H
a4	-	-	-	-
a5	-	-	-	-
a6	-	-	-	-
a8	8.35	8.49 (s) 1H	8.35	8.49 (s) 1H

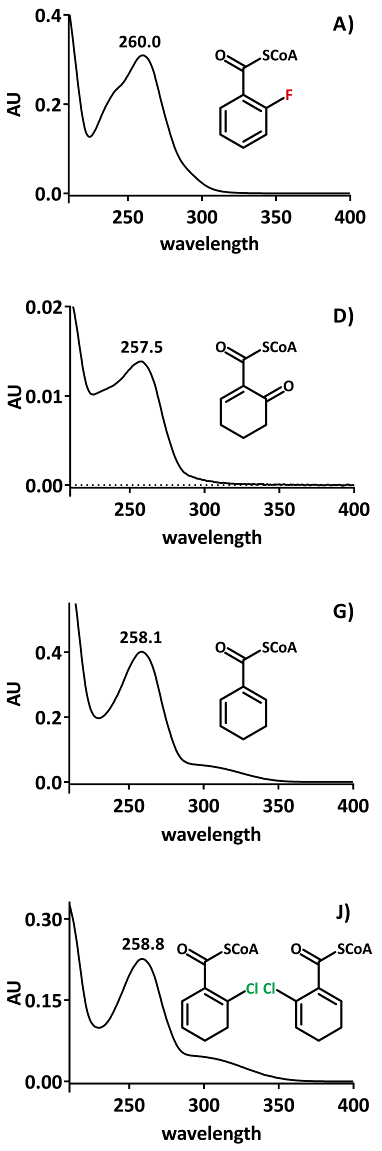

Supplementary Figure 1. UV/vis spectra of reaction products observed by UPLC analyses. Absorption maxima are indicated for **(A)** 2-F-BzCoA, **(B)** 2-F-/6-F-1,5-dienoyl-CoA, **(C)** 2-F-6-OH-1-enoyl-CoA, **(D)** 6-oxo-1-enoyl-CoA, **(E)** 3-OH-pimeloyl-CoA, **(F)** acetyl-CoA, **(G)** 1,5-dienoyl-CoA, **(H)** 6-OH-1-enoyl-CoA, **(I)** 2-Cl-BzCoA, **(J)** Cl-1,5-dienoyl-CoA.

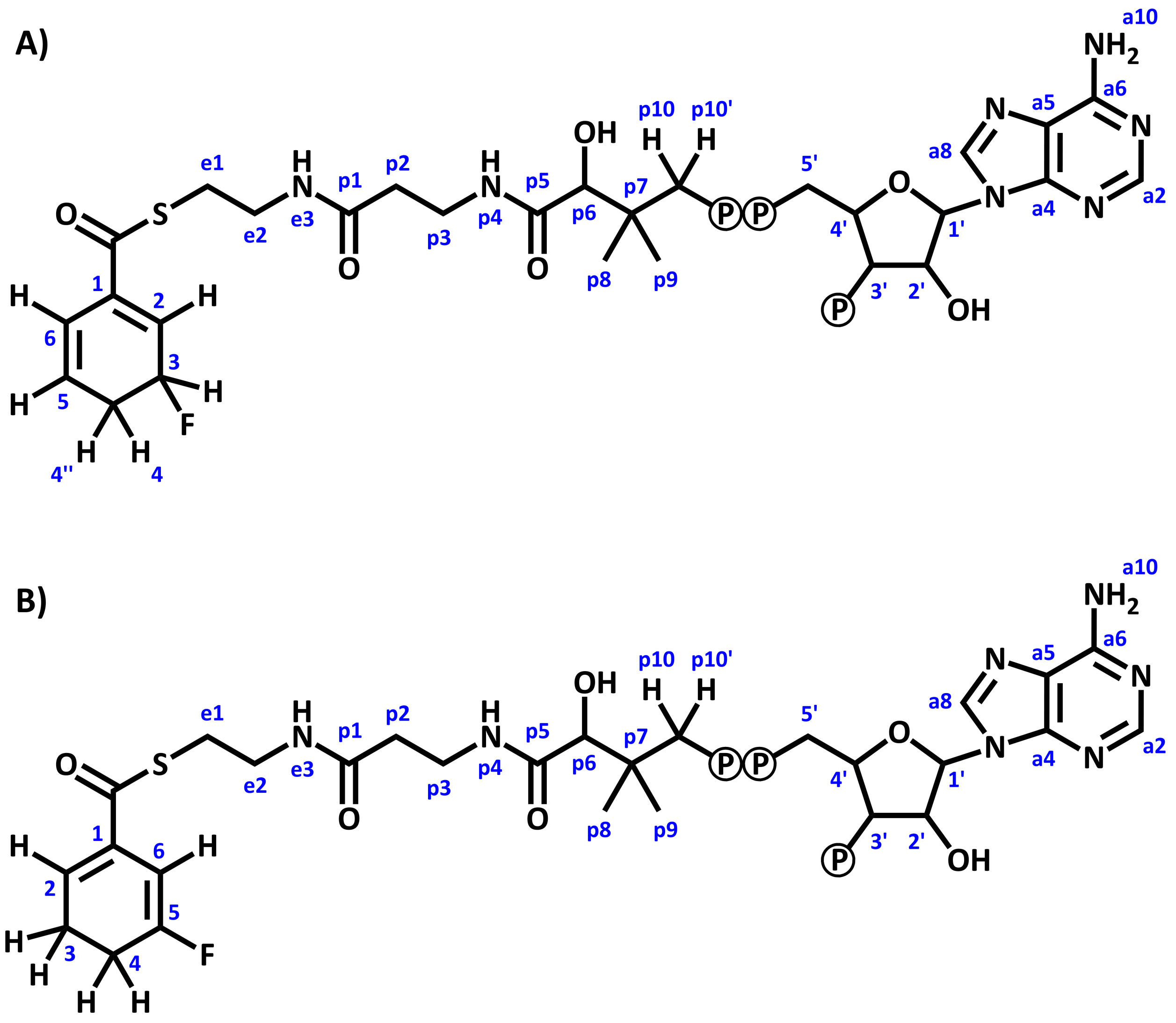

Supplementary Figure 2. Chemical structures of (A) 3-F-1,5-dienoyl-CoA and (B) 5-F-1,5-dienoyl-CoA, expected products of the reduction of 3-F-BzCoA by class I BCR. Positions with expected chemical shifts in ^{1}H NMR analyses have blue labels, which refer to chemical shifts shown in Supplementary Table 2.

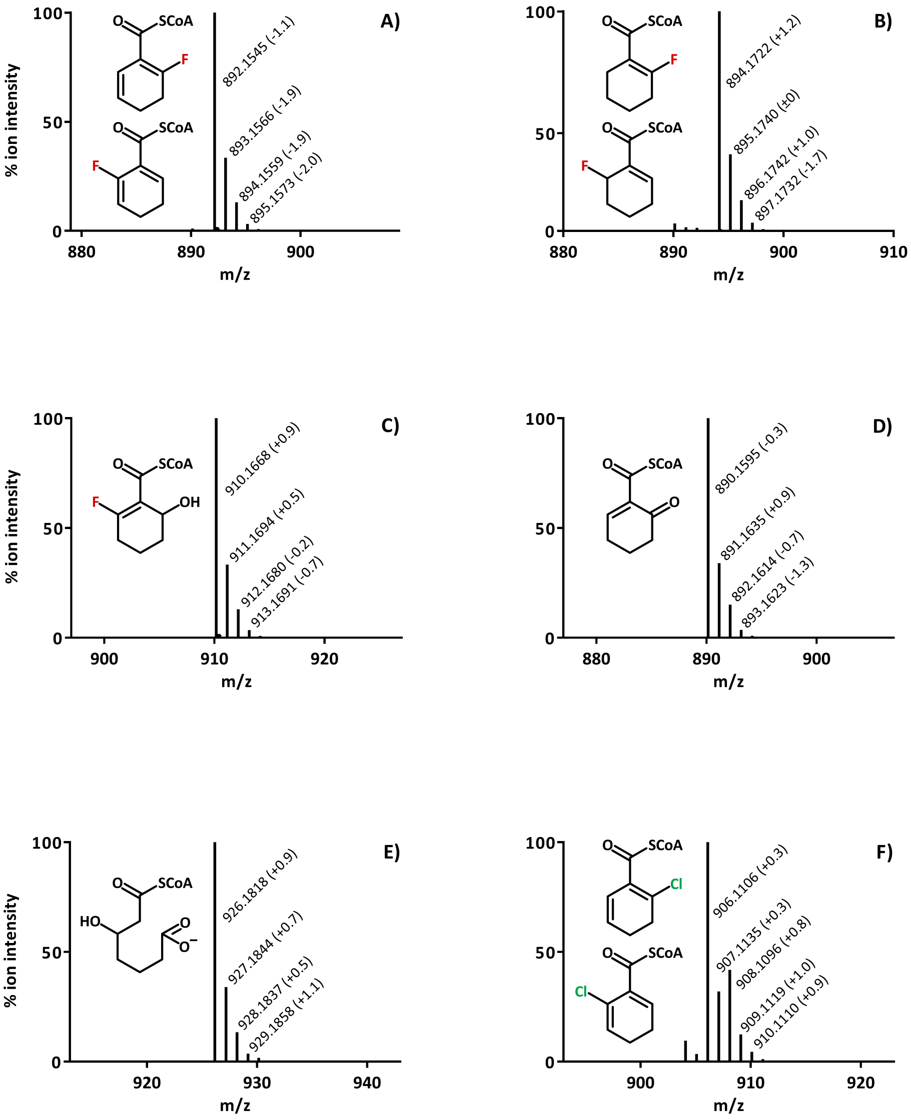

Supplementary Figure 3. Mass spectra of reaction products identified by ESI/Q-TOF-MS analyses. (A) 2-F-/6-F-1,5-dienoyl-CoA, **(B)** 2-F/6-F-1-enoyl-CoA, **(C)** 2-F-6-OH-1-enoyl-CoA, **(D)** 6-oxo-1-enoyl-CoA, **(E)** 3-OH-pimeloyl-CoA, **(F)** Cl-1,5-dienoyl-CoA. For each isotopic composition experimental m/z-values of the $[M+H]^+$ ions are given with ppm deviations from the theoretical values in parentheses.

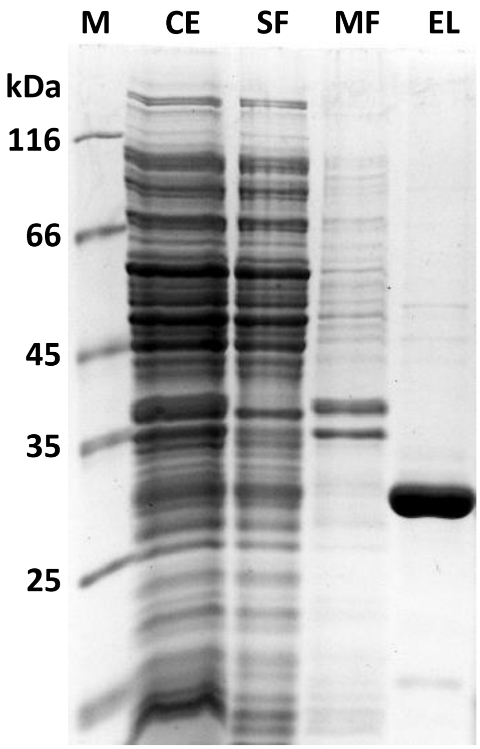

Supplementary Figure 4. SDS PAGE of heterologously produced DCH enriched by Ni-affinity chromatography. Different protein fractions obtained during the enrichment process were analyzed. Lanes are labeled correspondingly: M = protein MW marker, CE = crude extract, SF = soluble fraction (150,000×*g* supernatant), MF = membrane fraction (150,000×*g* pellet), EL = elution fraction (250 mM imidazole), with recombinant DCH (29.3 kDa) as the predominant protein component.

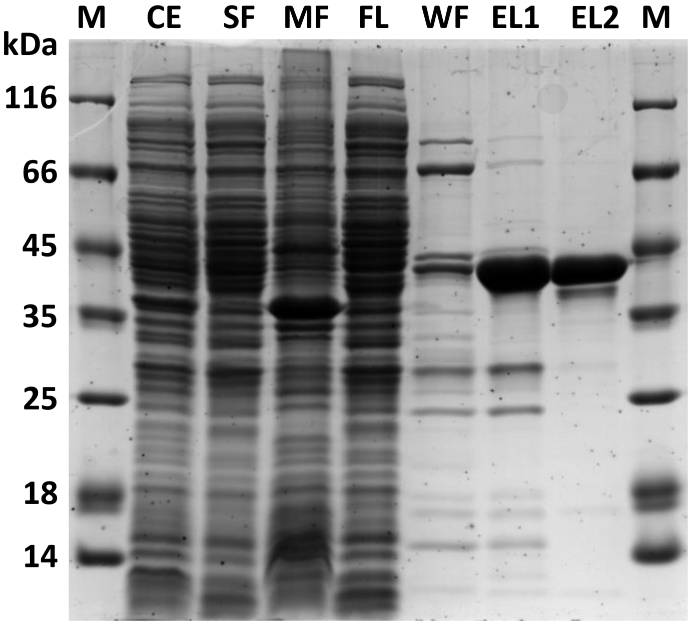

Supplementary Figure 5. SDS PAGE of heterologously produced OAH enriched by Ni-affinity chromatography. Different protein fractions obtained during the enrichment process were analyzed. Lanes are labeled correspondingly: M = protein MW marker, CE = crude extract, SF = soluble fraction (150,000×*g* supernatant), MF = membrane fraction (150,000×*g* pellet), FL and WF = flow through and wash fraction (60 mM imidazole), respectively, of affinity chromatography; EL1 and EL2 = elution fractions (120 and 250 mM imidazole) containing recombinant OAH (43.8 kDa) as the predominant protein component. EL1 and EL2 were pooled.

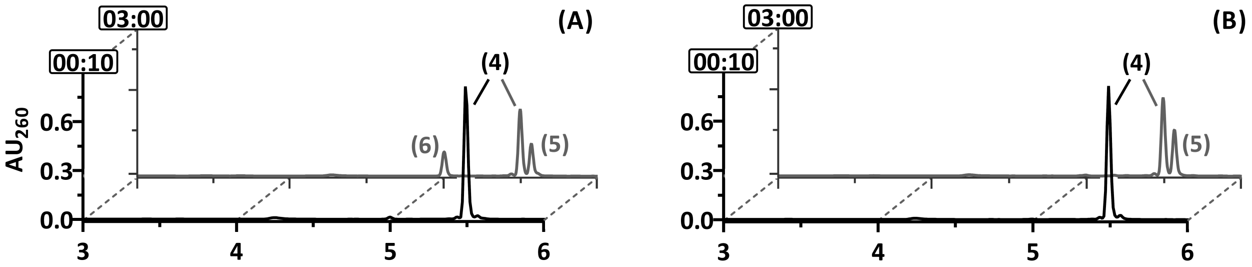

Supplementary Figure 6. UPLC diagrams demonstrating the time-dependent conversion of BzCoA by (A) BCR+DCH and (B) BCR+OAH. UPLC analyses of samples are shown that were taken at representative time points as indicated by the numbers (min) at the y-axis. Peaks of CoA thioester compounds are numbered as follows: BzCoA (4), 1,5-dienoyl-CoA (5), 6-OH-1-enoyl-CoA (6).
